# Comparison of two endogenous biomarkers of CYP3A4 activity in a drug–drug interaction study between midostaurin and rifampicin

**DOI:** 10.1007/s00228-014-1675-0

**Published:** 2014-05-21

**Authors:** Catherine Dutreix, Sebastien Lorenzo, Yanfeng Wang

**Affiliations:** 1 Novartis Pharma AG Basel, Basel, Switzerland; 2 Novartis Pharmaceuticals, East Hanover, NJ USA; 3 Present Address: Isis Pharmaceuticals, Carlsbad, CA USA

**Keywords:** Midostaurin, Rifampicin, CYP3A4 biomarker, 4β-hydroxycholesterol, 6β-hydroxycortisol to cortisol ratio

## Abstract

**Purpose:**

Midostaurin, a multitargeted tyrosine kinase inhibitor, is primarily metabolized by CYP3A4. This midostaurin drug–drug interaction study assessed the dynamic response and clinical usefulness of urinary 6β-hydroxycortisol to cortisol ratio (6βCR) and plasma 4β-hydroxycholesterol (4βHC) for monitoring CYP3A4 activity in the presence or absence of rifampicin, a strong CYP3A4 inducer.

**Methods:**

Forty healthy adults were randomized into groups for either placebo or treatment with rifampicin 600 mg QD for 14 days. All participants received midostaurin 50 mg on day 9. Midostaurin plasma pharmacokinetic parameters were assessed. Urinary 6βCR and plasma 4βHC levels were measured on days 1, 9, 11, and 15.

**Results:**

Both markers remained stable over time in the control group and increased significantly in the rifampicin group. In the rifampicin group, the median increases (vs day 1) on days 9, 11, and 15 were 4.1-, 5.2-, and 4.7-fold, respectively, for 6βCR and 3.4-, 4.1-, and 4.7-fold, respectively, for 4βHC. Inter- and intrasubject variabilities in the control group were 45.6 % and 30.5 %, respectively, for 6βCR, and 33.8 % and 7.5 %, respectively, for 4βHC. Baseline midostaurin area under the concentration–time curve (AUC) correlated with 4βHC levels (*ρ* = −0.72; *P* = .003), but not with 6βCR (*ρ* = 0.0925; *P* = .6981).

**Conclusions:**

Both 6βCR and 4βHC levels showed a good dynamic response range upon strong CYP3A4 induction with rifampicin. Because of lower inter- and intrasubject variability, 4βHC appeared more reliable and better predictive of CYP3A4 activity compared with 6βCR. The data from our study further support the clinical utility of these biomarkers.

**Electronic supplementary material:**

The online version of this article (doi:10.1007/s00228-014-1675-0) contains supplementary material, which is available to authorized users.

## Introduction

Cytochrome P450 3A4 (CYP3A4), the most abundant human CYP isoform [1], is involved in the metabolism of approximately half of all marketed drugs [2]. However, there is large intersubject variability in the expression and activity of CYP3A4, resulting from both genetic and nongenetic factors [3]. Sensitive probes such as midazolam are often used as exogenous markers to assess the in vivo activity of CYP3A4 [4, 5]. In contrast to exogenous markers, urinary 6β-hydroxycortisol to cortisol ratio (6βCR) and plasma 4β-hydroxycholesterol (4βHC) levels are endogenous biomarkers of CYP3A4 activity [6–14]. Indicative of cortisol and cholesterol metabolism by CYP3A4, respectively, urinary 6βCR and plasma 4βHC rise with increasing CYP3A4 activity [13, 15]. Besides being endogenous, these biomarkers are measured less invasively or noninvasively, making them attractive candidate markers for studies that involve monitoring pharmacokinetics (PK) and pharmacodynamics at multiple time points.

Midostaurin (PKC412; N-benzoylstaurosporin), a multitargeted tyrosine kinase inhibitor with activity in acute myeloid leukemia [16] and advanced systemic mastocytosis [17–19], is a sensitive CYP3A4 substrate [20]. Previously, we assessed plasma 4βHC level and urinary 6βCR in the rifampicin induction part of a drug–drug interaction study [20]. The goals of this analysis were to further evaluate the dynamic range of these biomarkers upon strong induction with rifampicin and to compare and evaluate whether these biomarkers can serve as covariates to explain intersubject variability of midostaurin pharmacokinetics in a clinical setting.

## Methods

### Study population and design

**Fig. 1:**
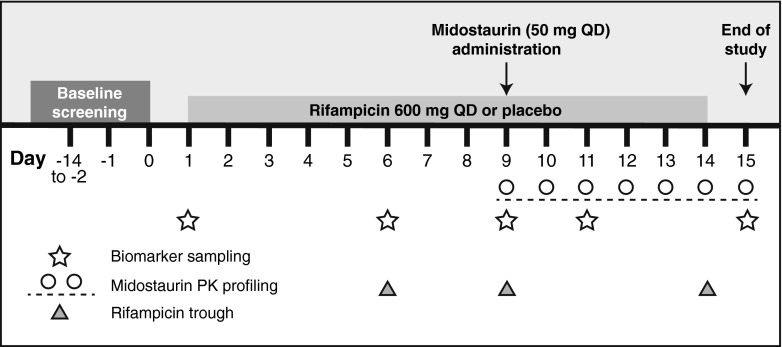
Study design. *QD* once daily, *PK* pharmacokinetic

The study population and study design have been reported previously [20]. Briefly, healthy adults aged 18 to 55 years weighing 50 to 90 kg and with a body mass index (BMI) of 18 to 29.9 kg/m^2^ were randomized 1:1 to receive placebo or rifampicin 600 mg once daily in the evening on days 1 through 14 (Fig. 1; Electronic Supplementary Material [ESM]-Methods). All subjects received midostaurin 50 mg on day 9.

### Pharmacokinetics and biomarker assessments

As described previously [20], a validated liquid chromatography/tandem mass spectrometry (LC-MS/MS) assay was used to assess midostaurin, rifampicin, and 4βHC levels in plasma and 6β-hydroxycortisol and cortisol levels in urine. Missing values were not imputed, and analyte concentrations below the lower limit of quantitation were treated as zero values. Additional details are reported in the ESM-Methods.

### Statistical analysis

The 4βHC concentrations and 6βCR were log-transformed and analyzed for each treatment group (placebo, rifampicin) with a linear mixed-effects model with visit (days 1, 9, 11, and 15) as fixed effect and subject as random effect. Point estimates and a corresponding 90 % CI of differences in visits were computed and antilogged to provide the GMR and 90 % CI of change in 4βHC and 6βCR (day 9/day 1, day 11/day 1, and day 15/day 1) by treatment group. The intersubject and intrasubject variabilities of 4βHC and 6βCR for each treatment were provided using the linear mixed-effect models. The correlation between the area under the concentration–time curve from time zero to infinity (AUC_inf_) of midostaurin and biomarkers (4βHC and 6βCR) was investigated with the Pearson correlation coefficient by treatment group.

## Results

### Baseline characteristics

Baseline characteristics in the PK population (*N* = 40, 20 in each arm) were balanced between study arms (Table 1). Most participants were male (60.0 %), and the majority were white (95.0 %). Median age, weight, and BMI were 44 years, 78.2 kg, and 24.9 kg/m^2^, respectively.

**Table 1:** Baseline demographics of the pharmacokinetics population

	Midostaurin + Rifampicin	Midostaurin + Placebo	All Participants
	(*n* = 20)	(*n* = 20)	(*N* = 40)
Median age (range), y	40 (23–52)	46 (30–53)	44 (23–53)
Male, *n* (%)	12 (60.0)	12 (60.0)	24 (60.0)
White, *n* (%)	19 (95.0)	19 (95.0)	38 (95.0)
Median weight (range), kg	77.8 (55–89)	78.3 (57–89)	78.2 (55–89)
Median BMI (range), kg/m^2^	24.5 (21–29)	25.1 (20–30)	24.9 (20–30)

*BMI* body mass index

### 4βHC levels and 6βCR

**Fig. 2:**
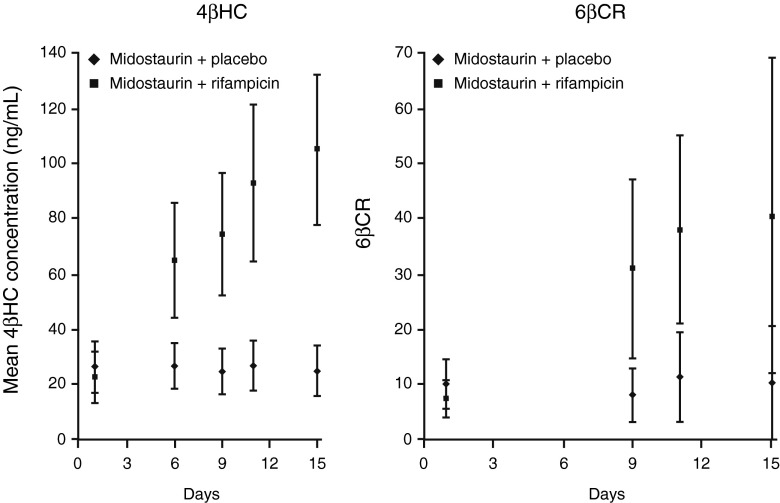
Plasma 4βHC levels and 6βCR over time in both the control (midostaurin + placebo) and treatment (midostaurin + rifampicin) groups (arithmetic mean ± SD). *4βHC* 4β-hydroxycholesterol, *6βCR* 6β-hydroxycortisol to cortisol ratio

Evidence of CYP3A4 induction and midostaurin PK are discussed in the ESM-Results. At baseline (day 1), 4βHC showed an intersubject variability of approximately 36 % in the midostaurin + rifampicin group and approximately 34 % in the midostaurin + placebo group. In the presence of rifampicin, the geometric mean estimate (90 % CI) of plasma 4βHC concentration in the midostaurin + rifampicin arm showed increases of 3.4-fold (3.2–3.6), 4.1-fold (3.8–4.3), and 4.6-fold (4.4–5.0) between day 1 and days 9, 11, and 15, respectively; variability ranged from 26 % to 29 % (Table 2; Fig. 2). In the midostaurin + placebo arm, the plasma 4βHC concentrations remained stable over time, as did intersubject variability (geometric CV% ≈ 36 %). Based on similar 4βHC levels in the placebo group, the intrasubject variability was estimated to be 7.5 %. There were no significant differences in 4βHC levels between males and females in either study arm (ESM-Supplemental Table 1).

**Table 2:** Changes in biomarker levels over time in each treatment arm

	Midostaurin + Rifampicin			Midostaurin + Placebo		
	(*n* = 20)			(*n* = 20)		
	Geometric Mean (CV%)	Range	Fold Increase (90 % CI)	Geometric Mean (CV%)	Range	Fold Increase (90 % CI)
4βHC, ng/mL						
Day 1	22.03 (36.45)	14.1–59.2	1.0 (baseline)	25.33 (34.25)	12.3–54.5	1.0 (baseline)
Day 9	74.35 (27.17)	55.5–152.0	3.4 (3.15–3.61)	23.38 (34.60)	12.5–48.5	0.9 (0.89–0.96)
Day 11	89.46 (29.47)	59.6–183.0	4.1 (3.79–4.35)	25.43 (33.85)	12.9–50.9	1.0 (0.97–1.04)
Day 15	102.70 (25.54)	69.5–178.0	4.6 (4.36–4.99)	23.28 (36.27)	11.9–53.0	0.9 (0.88–0.96)
6βCR						
Day 1	6.83 (47.75)	2.46–13.31	1.0 (baseline)	9.22 (46.64)	4.85–20.00	1.0 (baseline)
Day 9	27.73 (56.80)	10.10–75.20	4.1 (3.42–4.82)	6.92 (57.03)	3.38–21.90	0.8 (0.64–0.88)
Day 11	35.42 (53.63)	12.83–85.91	5.2 (4.37–6.15)	9.21 (50.81)	5.89–41.03	1.0 (0.85–1.17)
Day 15	32.14 (83.58)	11.46–117.82	4.7 (3.96–5.58)	7.49 (70.58)	3.45–53.13	0.8 (0.69–0.95)

4*βHC* 4β-hydroxycholesterol, 6*βCR* 6β-hydroxycortisol to cortisol ratio, *CV*% percent coefficient of variation

The intersubject variability at baseline was slightly higher for 6βCR (≈48 %) than for 4βHC (≈36 %). In the presence of rifampicin, the geometric mean estimate (90 % CI) of 6βCR showed 4.1-fold (3.4–4.8), 5.2-fold (4.4–6.2), and 4.7-fold (4.0–5.6) increases between day 1 and days 9, 11, and 15, respectively. The variability remained high in the rifampicin treatment group. In the placebo arm, the 6βCR values remained stable over time despite a high variability on day 15. Based on the repeated measurements in the placebo group, the intrasubject variability for 6βCR was estimated to be 30.5 %.

### Correlation between midostaurin AUC and CYP3A4 biomarker levels at baseline

**Fig. 3:**
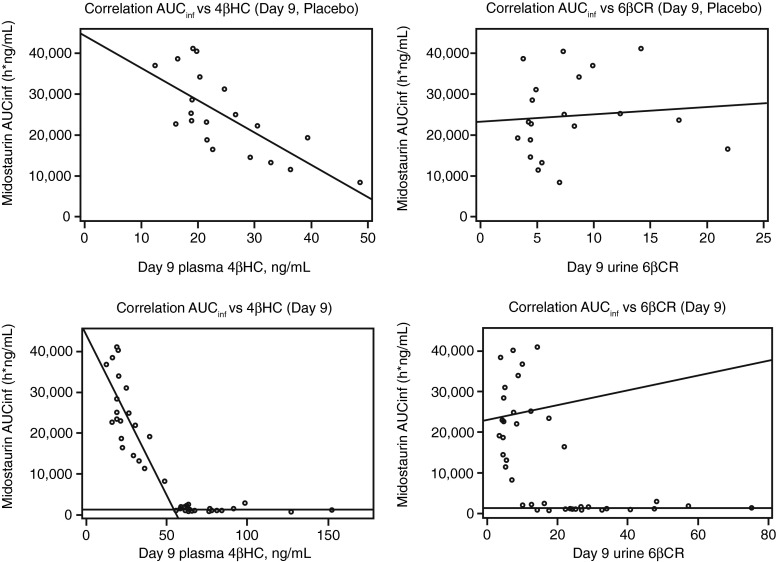
Correlation between midostaurin AUC_inf_ and 4βHC levels or 6βCR (day 9) in the placebo control group (upper panel) and in the placebo control plus rifampicin treatment groups combined (lower panel). *4βHC* 4β-hydroxycholesterol, *6βCR* 6β-hydroxycortisol to cortisol ratio, *AUC*_*inf*_ area under the concentration–time curve from time zero to infinity

In the placebo arm, midostaurin AUC correlated well with 4βHC levels at baseline (*ρ* = −0.72; *P* = .0003), but not with 6βCR at baseline (*ρ* = 0.0925; *P* = .6981; Fig. 3). In the pooled dataset that included the placebo and rifampicin groups, a clear separation of plasma 4βHC concentrations was observed between participants in the midostaurin + rifampicin arm (>55 ng/mL in all participants) and those in the midostaurin + placebo arm (≤55 ng/mL in all participants) after induction (Table 2). Considering all samples from days 9, 11, and 15, plasma 4βHC concentrations were higher in participants in the rifampicin arm (range, 55.5 ng/mL–183.0 ng/mL) than in those in the placebo arm (range, 11.9 ng/mL–53.0 ng/mL). However, for urinary 6βCR, pooled data showed significant overlap between the rifampicin and placebo groups (ranges 10.10–117.82 and 3.38–53.13, respectively), likely due to the large observed variability of the urine biomarker.

## Discussion

Both 6βCR and 4βHC level are well-known endogenous biomarkers for CYP3A4 activity [6–14]. CYP3A4/5 catalyzes the formation of 6β-hydroxycortisol from cortisol, both of which are excreted in urine [6]. Single-spot urine collection can be used to measure 6βCR [8, 15]. CYP3A4/5 also catalyzes the formation of 4βHC, which is formed from cholesterol [21]. Recent studies suggest that both 6βCR and 4βHC are viable and sensitive biomarkers for CYP3A4 activity; both showed good correlation with changes of the exogenous sensitive probe substrate midazolam when it was coadministered with rifampicin or ketoconazole [12, 14].

In the current study, healthy volunteers were administered a clinically relevant dose of rifampicin to induce CYP3A4 activity. CYP3A4 induction was associated with a notable increase in urinary 6βCR and plasma 4βHC concentrations, demonstrating that both 6βCR and 4βHC level can be used to monitor CYP3A4 activity. Levels of 4βHC had lower intersubject and intrasubject variability than 6βCR did, consistent with the long half-life of 4βHC in humans (17 days) [9]. Because 4βHC level is less variable within the sample subject, it can serve as a reliable biomarker for the baseline level of CYP3A4 activity in vivo. Midostaurin is a sensitive substrate of CYP3A4, as shown by the 94 % drop in AUC in the presence of rifampicin and a more than 10-fold increase with ketoconazole [20]. A high correlation coefficient of *ρ* = −0.72 between midostaurin AUC and 4βHC level suggests that a large portion (52 %) of the PK variability for midostaurin could be explained by CYP3A4 variability as reflected by different 4βHC levels. For drugs less sensitive to CYP3A4 metabolism, the correlation is likely to be less significant. The PK exposure–biomarker correlation analysis provides an added value of measuring baseline levels of 4βHC for drugs metabolized primarily by CYP3A4 in clinical studies. Additionally, prior work showed that 4βHC level was higher in women than in men [22]; while our data showed a similar trend, the differences were not significant.

While there was higher inter- and intrasubject variability in urinary 6βCR compared with plasma 4βHC levels, CYP3A4 induction was demonstrated more quickly with 6βCR than with 4βHC level. Urinary 6βCR increased 4.1-fold by day 9, close to the average plateau range between days 11 and 15, whereas the levels of 4βHC showed a continued increase between days 9 and 15, apparently due to its long half-life as discussed above. Although both cortisol and 6β-hydroxycortisol have a diurnal effect, their ratio remains stable over time [15, 23]. A steady state can be reached rather rapidly because of the short half-life of cortisol and its metabolite (approximately 1 h) [24], with little delay or lag time behind the changes of CYP3A4 activity in vivo. Thus, 6βCR and 4βHC may complement each other as CYP3A4 biomarkers. If a stable biomarker is needed, 4βHC would be the first choice. However, if a more rapid biomarker is necessary, 6βCR would be the marker of choice. If the outcome is unknown, as for new molecular entities, using both biomarkers in clinical studies would be recommended. Further studies may be warranted to evaluate whether the variability of 6βCR can be reduced or better managed.

## Electronic supplementary material

Below is the link to the electronic supplementary material.

ESM 1 (DOCX 30 kb)
