# Supplementary material for: Comparison of two endogenous biomarkers of CYP3A4 activity in a drug–drug interaction study between midostaurin and rifampicin
Source: Eur J Clin Pharmacol. 2014 May 21;70(8):915–20. doi: 10.1007/s00228-014-1675-0 (PMC4088993; doi:10.1007/s00228-014-1675-0)
Supplement: Supplementary file 1 — (DOCX 30 kb) [file 228_2014_1675_MOESM1_ESM.docx]

**Electronic Supplementary Material**

**SUPPLEMENTAL METHODS**

The study was conducted according to Good Clinical Practices and the ethical principles of the Declaration of Helsinki. Informed consent was obtained from each participant prior to randomization.

On days 1 through 8 and 10 through 14, participants fasted for 5 hours before and 2 hours after administration of placebo or rifampicin. On the morning of day 9, all volunteers received a single oral dose of midostaurin 50 mg, coadministered with placebo or rifampicin according to their randomization arm. Participants fasted at least 10 hours before to 3 hours after coadministration on day 9.

To assess rifampicin levels, predosing blood samples were collected on days 6, 9, and 14. The full profile of midostaurin pharmacokinetics was determined based on blood samples drawn prior to dosing and at several time points (0.5, 1, 1.5, 2, 3, 4, 6, 8, 12, 24, 36, 48, 72, 96, 120, and 144 hours) following midostaurin dosing on day 9. To assess urinary 6βCR, urine was collected in the morning on study days 1, 9, 11, and 15 during a 4-hour interval. Predose blood samples were drawn on the mornings of days 1, 9, 11, and 15 to determine plasma 4βHC concentration.

The urine biomarker was reported as a ratio of the levels of 6β-hydroxycortisol to cortisol (6βCR) measured in the same sample. Both 6β-hydroxycortisol and cortisol have short half-lives and have a diurnal effect when evaluated separately, but their ratio is relatively constant throughout the day [1-3]. Thus, 6β-hydroxycortisol levels were normalized by cortisol levels and 4-hour urine collection was considered adequate time to obtain information related to CYP3A4 activity. In contrast, both 4βHC and cholesterol have long half-lives of several days [4, 5], which makes plasma 4βHC stable over time [6]. Because normalizing 4βHC levels by cholesterol does not affect variability, concentrations of the plasma biomarker 4βHC were reported as measured. The assay used was specific to total (free and esterified) 4βHC and could differentiate 4βHC from 4αHC, which is metabolized by another enzyme, and other structurally related oxysterols. Additional information regarding the LC-MS/MS techniques used in this study have been previously reported [7].

**SUPPLEMENTAL RESULTS**

*Evidence of CYP3A4 induction and midostaurin PK*

Geometric mean rifampicin concentrations (geometric CV%) in the midostaurin + rifampicin arm were 12.0 ng/mL (106.9%), 12.7 ng/mL (73.0%), and 7.86 ng/mL (49.3%) on days 6, 9, and 14, respectively. In humans, midostaurin is metabolized by CYP3A4 into 2 major, pharmacologically active metabolites: CGP62221 (through O-demethylation) and CGP52421 (through 7-hydroxylation) [8-10]. The PK parameters for midostaurin and its 2 metabolites were reported in an earlier publication [7]. In the presence of rifampicin, the plasma AUC of midostaurin decreased by approximately 94% and half-life (t_1/2_) decreased 4.5-fold from 23 hours to 5 hours. A similar pattern was observed for CGP62221; its plasma AUC decreased by approximately 92% and t_1/2_ dropped from 34 hours to 7 hours. For CGP52421, the plasma AUC dropped by 75%, and t_1/2_ dropped by half from 175 hours to 87 hours; however, the change in CGP52421 exposure was likely underestimated because CGP52421 has a very long t_1/2,_ and the terminal phase was not fully captured within the limited sampling window designed for the study (mainly focusing on the parent drug rather than the metabolites).

**REFERENCES**

1. Lee C (1995) Urinary 6 beta-hydroxycortisol in humans: analysis, biological variations, and reference ranges. Clin Biochem 28:49-54

2. Tran JQ, Kovacs SJ, McIntosh TS, Davis HM, Martin DE (1999) Morning spot and 24-hour urinary 6 beta-hydroxycortisol to cortisol ratios: intraindividual variability and correlation under basal conditions and conditions of CYP 3A4 induction. J Clin Pharmacol 39:487-494

3. Weitzman ED, Fukushima D, Nogeire C, Roffwarg H, Gallagher TF, Hellman L (1971) Twenty-four hour pattern of the episodic secretion of cortisol in normal subjects. J Clin Endocrinol Metab 33:14-22

4. Chobanian AV, Burrows BA, Hollander W (1962) Body cholesterol metabolism in man. II. Measurement of the body cholesterol miscible pool and turnover rate. J Clin Invest 41:1738-1744

5. Diczfalusy U, Kanebratt KP, Bredberg E, Andersson TB, Bottiger Y, Bertilsson L (2009) 4beta-hydroxycholesterol as an endogenous marker for CYP3A4/5 activity. Stability and half-life of elimination after induction with rifampicin. Br J Clin Pharmacol 67:38-43

6. Kanebratt KP, Diczfalusy U, Backstrom T, Sparve E, Bredberg E, Bottiger Y, Andersson TB, Bertilsson L (2008) Cytochrome P450 induction by rifampicin in healthy subjects: determination using the Karolinska cocktail and the endogenous CYP3A4 marker 4beta-hydroxycholesterol. Clin Pharmacol Ther 84:589-594

7. Dutreix C, Munarini F, Lorenzo S, Roesel J, Wang Y (2013) Investigation of CYP3A4-mediated drug-drug interactions on midostaurin in healthy volunteers. Cancer Chemother Pharmacol 72:1223-1234

8. Levis M, Brown P, Smith BD, Stine A, Pham R, Stone R, Deangelo D, Galinsky I, Giles F, Estey E, Kantarjian H, Cohen P, Wang Y, Roesel J, Karp JE, Small D (2006) Plasma inhibitory activity (PIA): a pharmacodynamic assay reveals insights into the basis for cytotoxic response to FLT3 inhibitors. Blood 108:3477-3483

9. Yin OQ, Wang Y, Schran H (2008) A mechanism-based population pharmacokinetic model for characterizing time-dependent pharmacokinetics of midostaurin and its metabolites in human subjects. Clin Pharmacokinet 47:807-816

10. Wang Y, Yin OQ, Graf P, Kisicki JC, Schran H (2008) Dose- and time-dependent pharmacokinetics of midostaurin in patients with diabetes mellitus. J Clin Pharmacol 48:763-775

**Supplemental Table 1** Changes in 4βHC level over time by sex.

|  | **Midostaurin + Rifampicin** | | **Midostaurin + Placebo** | |
| --- | --- | --- | --- | --- |
|  | **Female**  **(n = 8)** | **Male**  **(n = 12)** | **Female**  **(n = 8)** | **Male**  **(n = 12)** |
| Geometric mean (CV%) 4βHC level, ng/mL | | | | |
| Day 1 | 24.32 (49.06) | 20.63 (26.20) | 21.84 (25.47) | 27.96 (36.49) |
| Day 9 | 78.66 (33.11) | 71.60 (23.32) | 19.68 (22.76) | 26.21 (36.84) |
| Day 11 | 100.40 (33.67) | 82.83 (24.72) | 21.02 (22.75) | 28.87 (34.31) |
| Day 15 | 110.95 (28.77) | 97.53 (22.94) | 19.73 (23.84) | 25.99 (39.53) |
| Geometric mean (CV%) 4βHC ratio vs day 1 | | | | |
| Day 9 | 3.23 (24.73) | 3.47 (16.90) | 0.90 (8.10) | 0.94 (14.12) |
| Day 11 | 4.13 (29.81) | 4.02 (23.29) | 0.96 (7.29) | 1.03 (17.28) |
| Day 15 | 4.56 (34.38) | 4.73 (23.00) | 0.90 (8.28) | 0.93 (11.93) |

4βHC, 4β-hydroxycholesterol; CV%, percent coefficient of variation
